# Engineering Protein Dynamics through Mutational Energy Landscape Traps

**DOI:** 10.1021/acs.jcim.4c01928

**Published:** 2025-01-08

**Authors:** Lucas de Almeida Machado, João Sartori, Paula Fernandes da Costa Franklin, Mauricio G. S. Costa, Ana Carolina Ramos Guimarães

**Affiliations:** † Instituto Nacional de Saúde da Mulher, da Criança e do Adolescente − Fiocruz, Rio de Janeiro, Brazil 22250-020; ‡ Laboratório de Genômica Aplicada e Bioinovações − Instituto Oswaldo Cruz/Fiocruz, Rio de Janeiro, Brazil 21040-900; § Programa de Computação Científica − Fiocruz, Rio de Janeiro, Brazil 21040-900; ∥ Programa de Pós-Graduação em Biologia Computacional e Sistemas − Instituto Oswaldo Cruz/Fiocruz, Rio de Janeiro, Brazil 21040-900

## Abstract

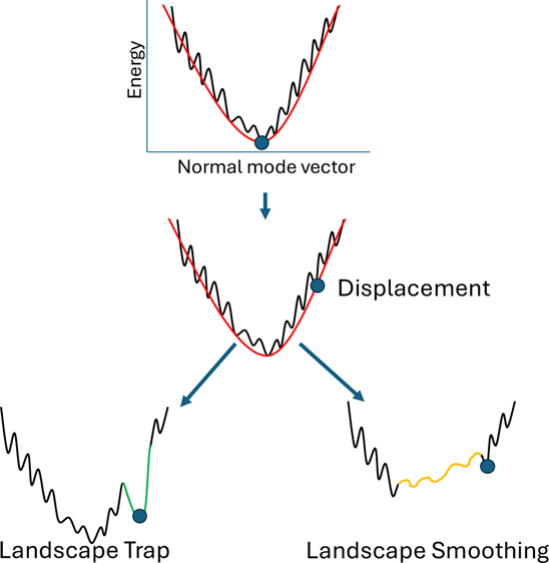

Protein dynamics is essential for various biological processes, influencing functions such as enzyme activity, molecular recognition, and signal transduction. However, traditional protein engineering methods often focus on static structures, lacking tools to precisely manipulate dynamic behaviors. Here, we developed Mutational Energy Landscape Trap (MELT), a novel method designed to control protein dynamics by combining Normal Mode Analysis (NMA) and *in silico* mutagenesis. MELT works by displacing protein structures along low-frequency normal modes and introducing mutations to either lock proteins in these conformations or increase dynamics along the chosen normal modes. We tested MELT using hen-egg lysozyme as a model system. The method was validated by monitoring relevant collective coordinates during molecular dynamics simulations and evaluation of the collective movements of each construct. Our experiments showed that MELT was able to consistently create new protein sequences with the desired dynamical behavior in simulations. It demonstrates its potential for applications in the field of protein engineering, being an unprecedented way of manipulating protein features.

## Background

Over the years, significant advancements have been made in understanding the relationship between protein sequence and structure, which has ultimately led to the development of a plethora of protein engineering methods.^1^ The design of new structures has proven to be a successful approach, as has recently seen its peak with the advent of diffusion methods and large language models.^2,3^ However, protein dynamics also plays an essential role in biological functions,^4,5^ and the current state-of-the-art engineering methods are not developed to directly manipulate dynamics. Therefore, engineering protein dynamics presents a unique challenge, because in principle it is difficult to predict how it is affected by mutations without performing long molecular dynamics (MD) simulations.

MD is a well-established method for studying protein dynamics using force fields that enable a proper representation of the molecular interactions and the surrounding environment. However, despite the advances observed in the last decades, reaching time scales of biological interest is still not feasible through MD, especially for supramolecular structures.^6^

A cost-effective approach to overcome this limitation is the application of analytical methods to study protein dynamics. Among these is normal-mode analysis (NMA), which is a computational technique that allows the characterization of intrinsic protein motions encoded by their folds.^7^ NMA requires a single structure for the calculations and it yields all possible motions decomposed in separate modes with their associated vibrational frequencies. Among the advantages of NMA, the fast and exact calculations giving access to the entire vibrational spectrum stand out. Usually, a few low-frequency modes describe the large amplitude motions correlated with biological functions.^8,9^ Moreover, despite the simplifications, there is a close correspondence between cooperative motions deducted by NMA and those obtained from long MD simulations, as exemplified in many applications.^10,11^

In this paper, we present Mutational Energy Landscape Sculpting (MELT), a novel approach for engineering protein dynamics by combining NMA and structure-based protein engineering. Conceptually, MELT’s goal is to modify the energy landscape through mutagenesis aiming to stabilize other conformational populations distinct from those observed in the wild-type form. To achieve this effect, we displace the structure following a relaxed low-frequency mode, and taking this displaced state as reference, we introduce mutations to maximize stability of this particular conformation. In theory, our approach can result in a trap in the energy landscape, assuring that the mutated protein stays in the displaced state, or a smoothing of the landscape, generating constructs with increased conformational plasticity, thus, sculpting the energy landscape in order to create structures that can inhabit different regions in conformational space.

We applied MELT to engineer the dynamic properties of hen-egg lysozyme. In this proof-of-concept study, we investigate how motions along a well-described lysozyme motion could be used to generate new designs with distinct dynamical behavior. Therefore, we selected the softest mode of motion obtained by NMA (mode 7, as the first six modes correspond to overall rotations and translations). Indeed, previous studies have already demonstrated that this lowest frequency mode accurately describes the lysozyme breathing motion.^12^ In principle, other directions could be selected, but in this first study, we opted to work with a validated protein motion. Here, we used the motions along mode 7 displacement vectors in both directions, since in principle both directions are equiprobable. Based on this assumption, we generated “open” and “closed” designs” by following the opposite directions of mode 7.

## Theory

Using Normal Mode Analysis (NMA) to explore protein dynamics facilitates the investigation of collective motions that are not easily accessible through conventional MD. Given a protein structure at an energy minimum (eq 1), defined by the 3D coordinates of the atoms of a protein structure from atom 1 to *n*, a set of 3*n*-6 nontrivial normal mode vectors (eq 2) can be calculated. The vectors can be used to generate a displaced structure along a normal mode (eq 3), where λ stands for scaling factor describing the magnitude of the displacement.
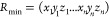
1
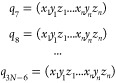
2
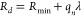
3

Now, taking the displaced structure *R*_*d*_, and minimizing its potential energy with minimal backbone displacement, we reach the local minimum *R*_*opt*_, which has atomic coordinates closer to *R*_*d*_ than to *R*_*min*_. It is then possible to optimize the wild-type protein sequence (represented by the amino acid vector *M*_*wt*_), to be better suited to the displaced coordinates by iterative mutagenesis, generating an engineered sequence (the amino acid vector *M*_*eng*_). Which can result in two scenarios: Energy landscape trap (eq 4) or Energy landscape smoothing (eq 5), where *U*(*R*_*opt*_|*M*_*wt*_) and *U*(*R*_*opt*_|*M*_*eng*_) are the potential energies at the displaced coordinates given the wild-type or the engineered sequences, respectively.
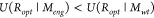
4
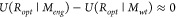
5

**Figure 1:**
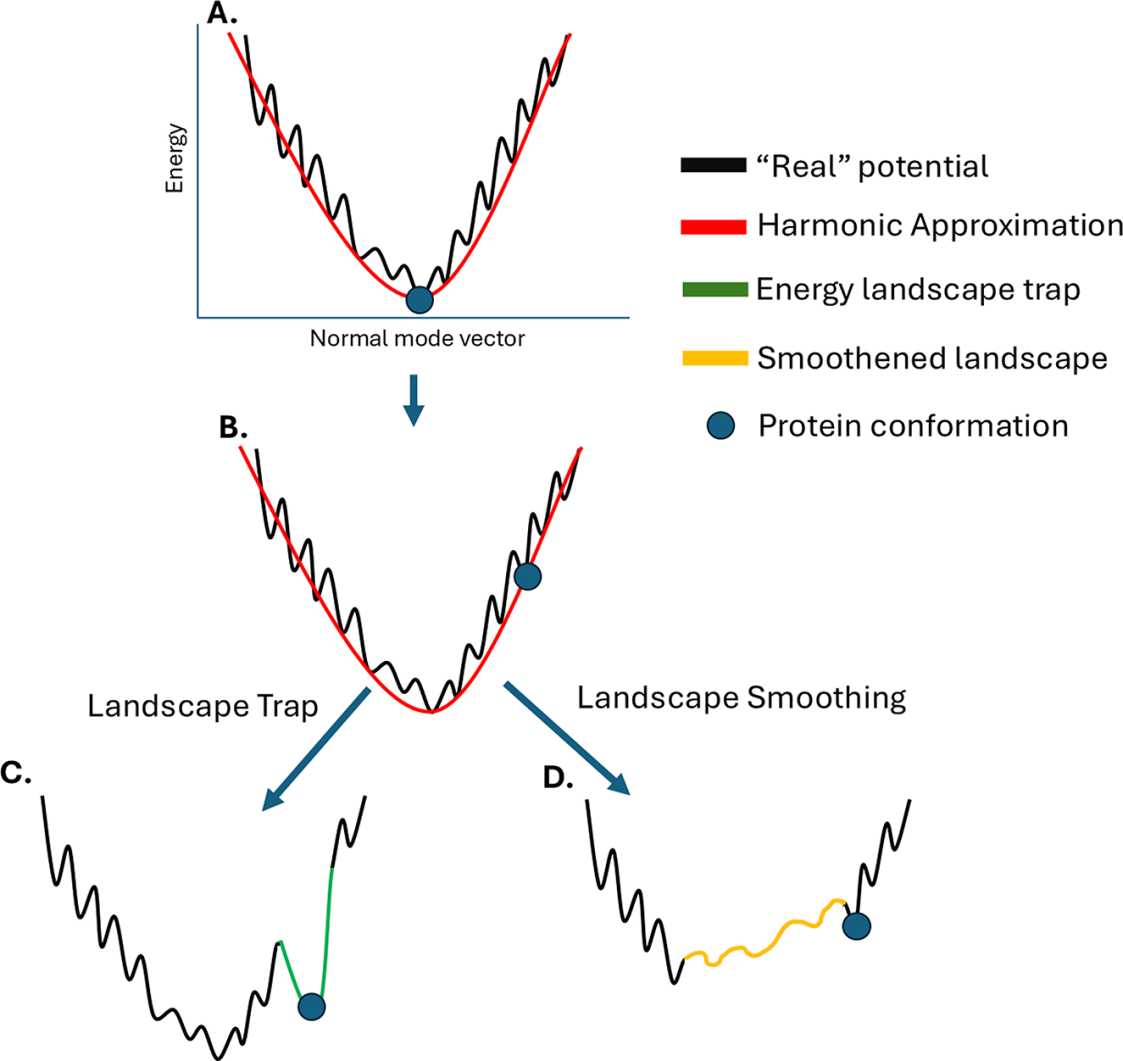
Rationale of the method. The scheme shows an idealized energy landscape with the Y axis representing the potential energy and the X axis the normal mode vectors. First, the normal mode vectors are calculated for a given protein conformation (A). Then, the structure is displaced along a selected NM vector (B), and finally, a combinatorial mutational scan is performed to identify sequence variations that stabilize the displaced conformation, potentially generating an energy landscape deformation. The procedure can result in two different outcomes: a deeper minimum (trap) corresponding to the displace coordinates (C) or smoothing of the landscape (D).

The MELT procedure involves the following steps: i. Calculating the Normal Modes of a given protein structure. ii. Displacing the protein along a set of selected normal mode vectors. iii. performing energy minimization on the displaced structure. IV. Optimizing the Δ*G*_fold_ of the displaced structure using pyRosetta’s design routine by mutating the original protein sequence. The rationale for the method is visually explained in Figure 1, where a given protein is displaced and then designed to better suit a given coordinate along a normal mode

In this study, the designed structures were tested using MD simulations, in order to verify whether the procedure is capable of changing protein dynamics in the simulation in a predictable manner and the relevant coordinates, cavity volumes, and frustration were measured to compare the behavior of the designs.

## Results and Discussion

We applied MELT to engineer the dynamic properties of hen-egg lysozyme. By leveraging NMA to identify low-frequency modes that govern large-scale conformational changes, we displaced the protein structure along mode 7 to generate displaced conformational states. These states were stabilized through mutagenesis using pyRosetta, optimizing the mutations to lock the protein in the displaced conformations or at least favor its conformational dynamics along mode 7. To quantify these changes, we monitored the distance between residues 47 and 109, which are key indicators of the hinge-bending motion essential for the enzyme’s activity. The resulting designs were named according to their conformational states: OP1 and OP2 for designs stabilized in the “open” state, and CL1 and CL2 for designs stabilized in the “closed” state. The results show that the engineered proteins displayed distinct and predictable dynamic behaviors, validating the effectiveness of MELT in controlling protein dynamics in MD simulations.

### Design Procedure and Sequence Analysis

**Figure 2:**
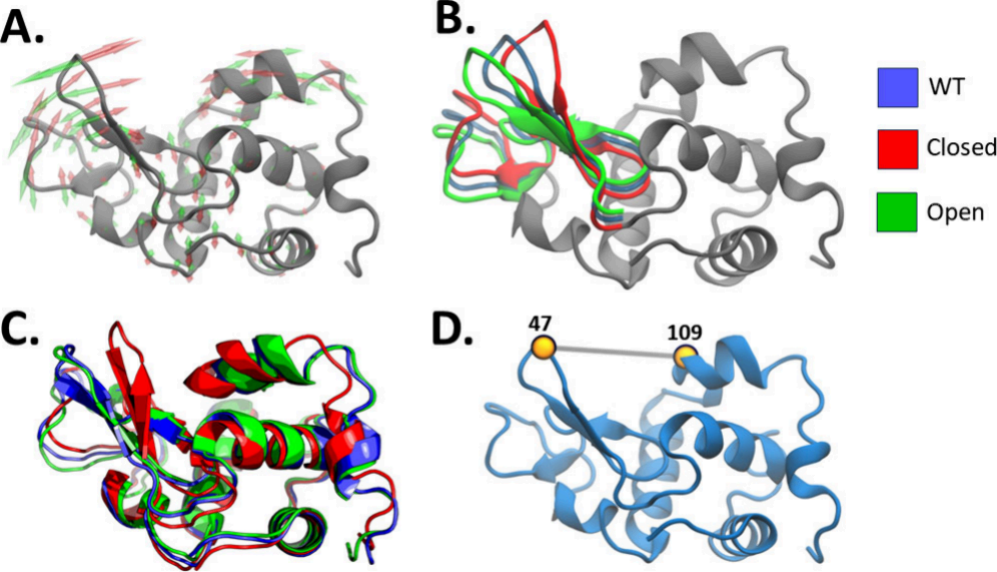
Structural representation of MELT’s procedure. (A) displays the vectors of mode 7 in both directions, where red arrows represent the closing motion, and green arrows the opening motion. (B) displays the structures of hen-egg lysozyme when displaced in both directions along the normal mode vectors. (C) depicts the relaxed designed structures compared to the WT. (D) showcases the distance between residues 47 and 109, which is used as a collective coordinate for probing the closing motion of the structure, represented in the structure as a bold gray line.

Figure 2A shows the directions of the hinge bending motion described by the lowest frequency normal mode. The independent 2 Å RMS displacements along the close and open directions lead to structural variations mostly on the L-domain, as reported in other studies^13^ (Figure 2B). An inspection of the relaxed designed structures (Figure 2C) shows that after the FastRelax, the OP structure returned to a conformational state very close to the WT structure, while the CL structure remained in a “closed state” similar to the geometrically displaced structure used as input for producing the designs. Given that linear displacements along normal modes lead to structural distortions, we evaluated whether the relaxation procedure carried out with Rosetta was sufficient to ensure the structural quality of the configurations considered as inputs for the design procedure. Table 1 shows low Molprobity scores for the relaxed structures associated with a dramatic decrease of the overall energy (in the scale of Rosetta Energy Units - REU). Therefore, the relaxation steps after the linear displacements fixes structural distortions leading to geometrically acceptable states for the protein design procedure. The degree of displacement after the FastRelax could explain the magnitude of the effect on the opening of each design, where the closed designs showed a more pronounced effect in the MD simulations.

**Table 1:** Structural Quality of Displaced Structures

**System**	**Rosetta Energy Units**	**MolProbity Score**	**RMSD in relation to crystal structure (Å)**
2LZT	–404.43	1.25	–
Displaced 2LZT (Open)	344.26	1.72	1.765
Displaced 2lZT (Closed)	377.61	1.53	1.765
Displaced 2LZT (Open) - Relaxed	–412.14	0.99	0.78
Displaced 2lZT (Closed) - Relaxed	–396.8	0.87	1.23

**Figure 3:**
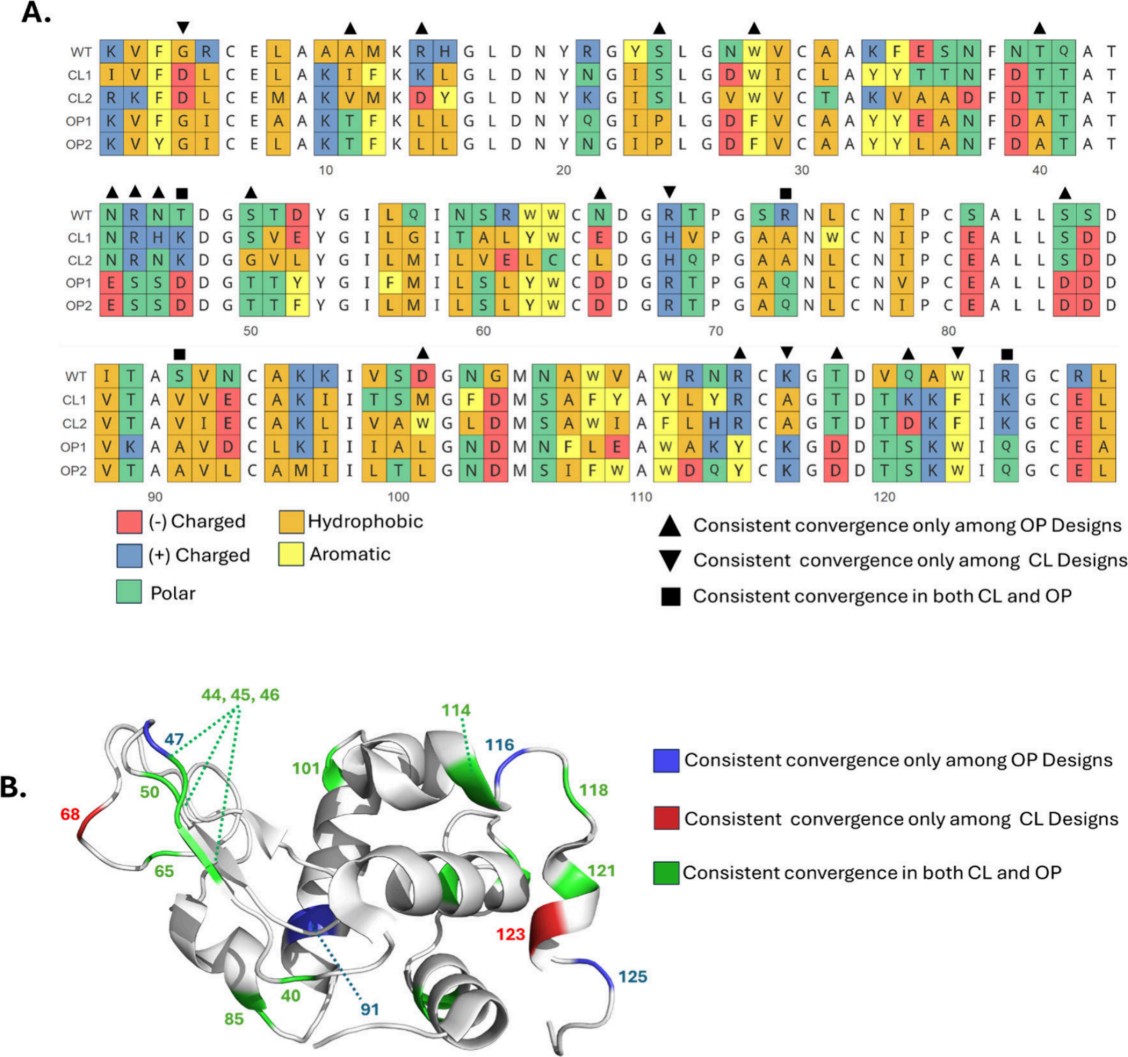
Sequence alignment of the WT sequence and all the designs and sequence convergence. (A) shows the multiple sequence alignment colored according to the physicochemical properties of the differing residues as given in the legend. The residues shown in the structure are marked above the alignment columns accordingly. (B) depicts the lysozyme structure highlighting the convergence profiles.

After the design procedure, some patterns could be observed in the new sequences (Figure 3). Residues 16–20 are conserved in all designs, indicating a possible optimal sequence fitness in this region. Several residues exhibit distinct substitutions in OP and CL designs. For instance, residue 47 is a Threonine in WT, but in both OP designs is replaced with an Aspartic acid, while in CL designs, it is substituted by a Lysine. Similarly, residue 73, which is an Arginine in the WT, becomes an Alanine or a Glutamine in CL and OP designs respectively. These examples show that some residue preferences are related to the conformational state of the template considered for the design.

On the other hand, similar physicochemical properties were observed in a few positions in both OP, and CL designs. Residue 81, originally a Serine in the WT, is consistently substituted by a charged glutamic acid in both OP and CL designs. Likewise, residue 86, which is a serine in the WT, is replaced by aspartic acid in all designs. Additionally, residue 128, an Arginine in the WT, is also consistently replaced by Glutamic acid in all designs. These similarities point to suboptimal regions in the WT sequence that do not mutate in a design-specific manner and are optimized in all new sequences. Interestingly, some of the positions that consistently converged to certain residues in the OP or CL designs are located in opposite ends of the cavity (residues 47 and 101) which explains the control over the closed state. Changes were also found in hinge regions (residues 47, 73, 65, 85, 116 and 118) which can also be related to the opening and closing of the cleft. The protein core was mostly undisturbed, probably because many core residues are optimal for all designs. Exclusive mutations were more consistently found in OP designs than in the CL designs. Interestingly, the β-sheet containing residues 44, 45, and 46 displays many mutations that are exclusive to OP designs, which indicates that some particular residues in this region favor the opening of the binding cleft.

### MELT Allows for Control of Relevant Collective Coordinates in MD Simulations

**Figure 4:**
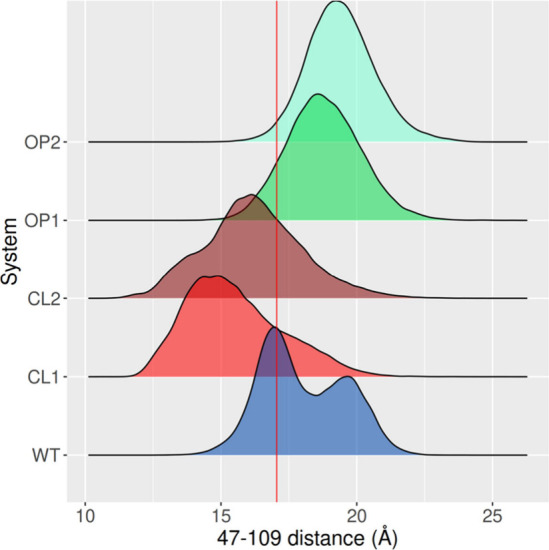
Distributions of the distance between residues 47 and 109. The distributions show a bimodal behavior for WT, while all closed designs explore lower values and both open designs superimpose with the more open states of the WT enzyme. The red line shows the distance value found in the crystal structure.

After performing MD for all systems considered in this study, the distributions of the distance between residues 47 and 109 were computed. In the WT system, a bimodal distribution was obtained with a dominant state where both residues are ∼17 Å apart, and a more open one where they are ∼20 Å apart (Figure 4). The conformational populations obtained for OP designs revealed unimodal distributions centered at open states with distance values around 20 Å. They likely stabilize conformers with higher opening amplitude, as the WT enzyme is also capable of exploring, although less frequently. Part of this behavior may be explained by the fact that the relaxed open designs come back to a similar state as the WT after the FastRelax procedure, as shown in Figure 2.

On the other hand, the CL designs mostly explored closed states with broader distributions and peaks on lower distance values than the dominant state obtained for the WT. Furthermore, CL1 exhibited substantial exploration of even closer states than CL2. It is plausible to rationalize these findings based on the observation that closed states are stabilized by an increase in attractive interactions between the opposite sides of the substrate-binding cleft, which ultimately generate an energy landscape trap that directly impacts on the dynamic equilibrium. Moreover, not just the distributions are visually very different, but statistical comparison between the of the distance distributions obtained for each design and the WT using Mann–Whitney U test, shows that all systems the distributions are significantly different from the WT (*P* < 2.2^–16^), confirming the efficacy of the method in manipulating the conformational dynamics of the protein in a predictable manner when evaluating the MD simulations.

### Designs Derived from Different OP and CL Structures Converge to Similar Flexibility Profiles

**Figure 5:**
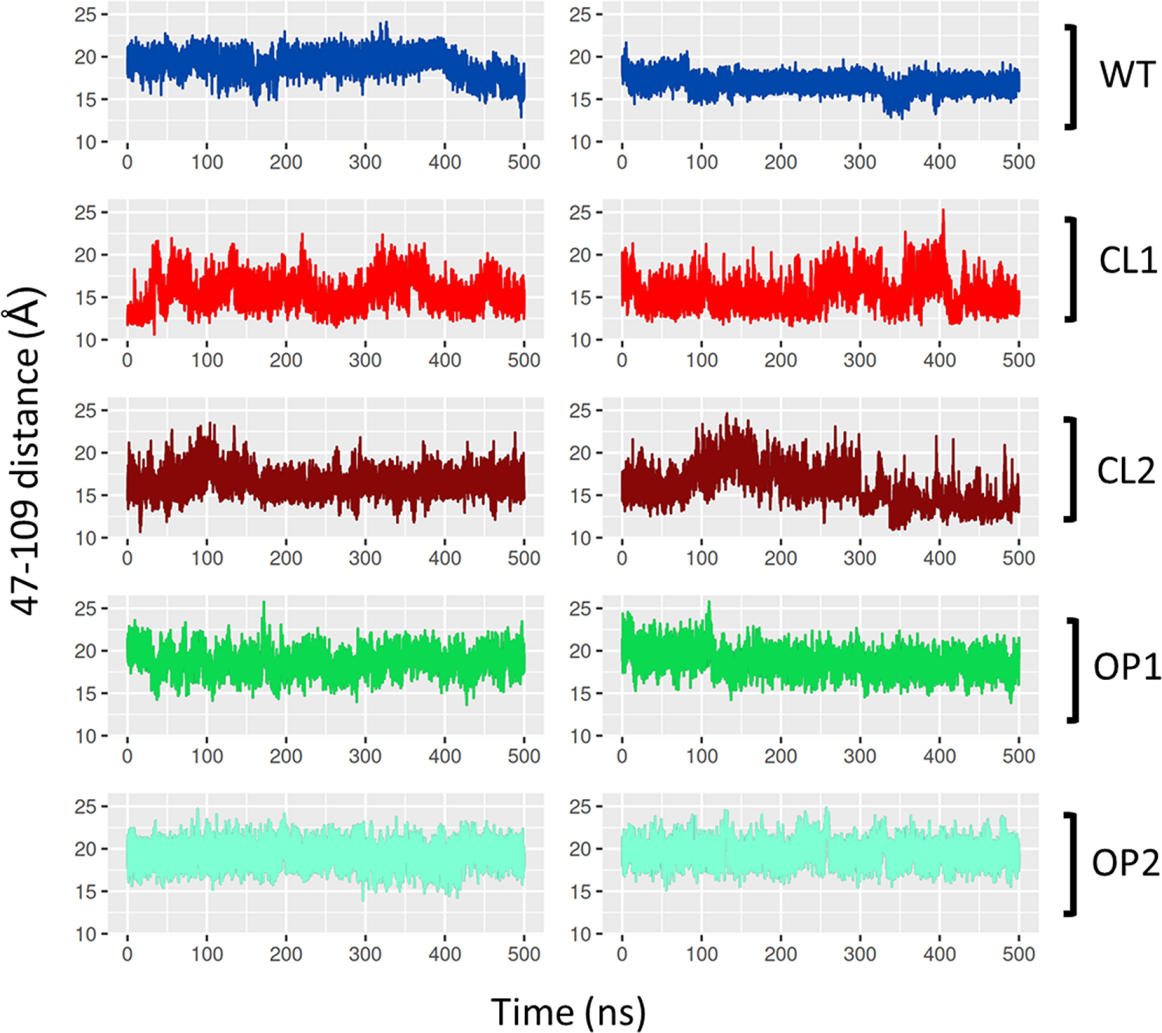
Time evolution of the distance between residues 47 and 109 during MD simulations. Each system is indicated at the right of each plot, both replicas of each system are shown.

The inspection of the distance between residues 47 and 109 in each simulation replica reveals distinct behaviors of the CL and OP designs (Figure 5). Both closed designs explored a large amplitude of distance values, oscillating between closed and open states, while the WT system exhibited oscillations with smaller magnitude. The OP designs, on the other hand, remained stable in a single state throughout the entire simulations.

**Figure 6:**
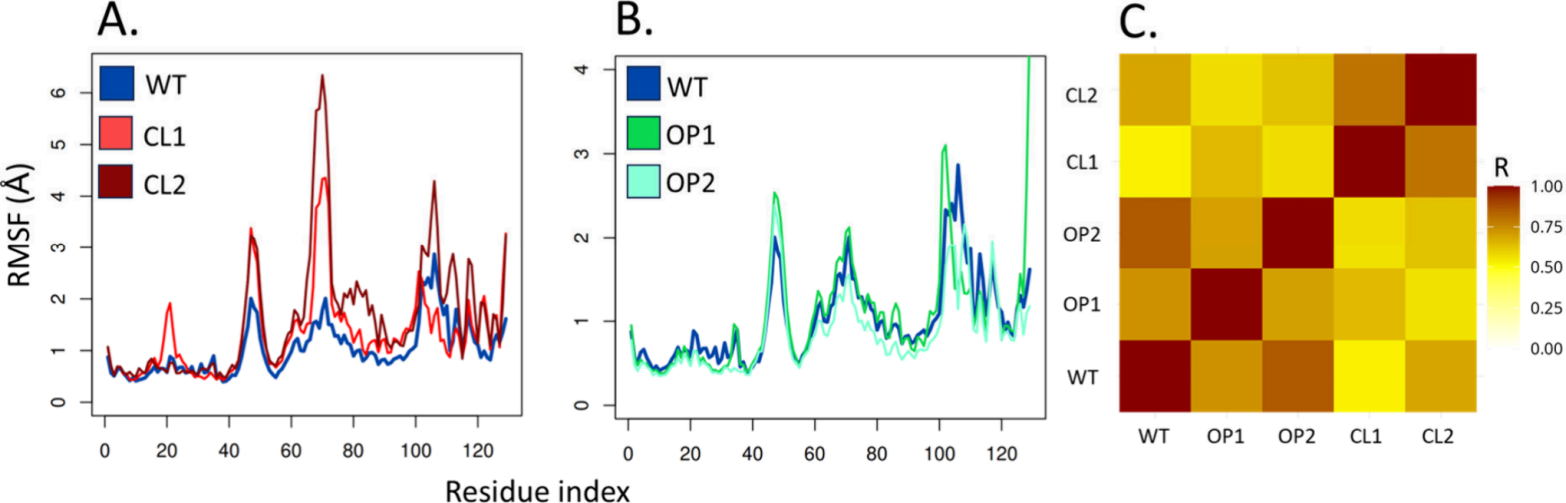
Root-mean-square fluctuation profiles of each simulated system. (A) The RMSF of the closed designs is compared to the WT. (B) The RMSF of the open designs is compared to the WT protein. Different scales were adopted to highlight small differences between the WT system and each design. (C) The correlation between the fluctuations of each pair of systems is shown.

Analysis of the RMSF profiles (Figure 6) highlights the differences between the designs. The RMSF peaks in CL1 and CL2 may be due to closing along the hinge-bending motion. The highest values are found in the loop region between residues 60–70, and the loop region 40–50 which is displaced along mode 7. CL2 also shows high fluctuations in the region 70–90 which connects both sides of the substrate-binding cleft, while CL1 has a peak in residues 20 and 21, which are distant from the catalytic site. The closed designs show only small differences in RMSF when compared to WT, mainly in residues 30–40, and are very similar to the original enzyme when it comes to fluctuations, which is interesting, since the open designs have higher 47–109 distances than the WT, confirming that the OP structures do not undergo large opening motions, but instead fluctuated slightly around an open structure. Notably, both the RMSF profiles of both OP designs exhibit strong correlation with the WT fluctuations. This observation could be due to the similarity between the relaxed OP structure and the crystal structure. On the other hand, the fluctuation profiles obtained for the CL1 and CL2 designs were highly similar to each other but clearly distinct from the others. Therefore, the design procedure using NMA-displaced structures not only allows for selecting a predominant conformational population during the simulations, but also consistently creates fluctuation patterns in constructs created from the same sets of displacement vectors, despite primary structure differences. In addition to the correlation between fluctuations, the active site cavities of the centroid structures obtained from the MD simulations of the WT and OP1 are also similar (Figure S1).

**Figure 7:**
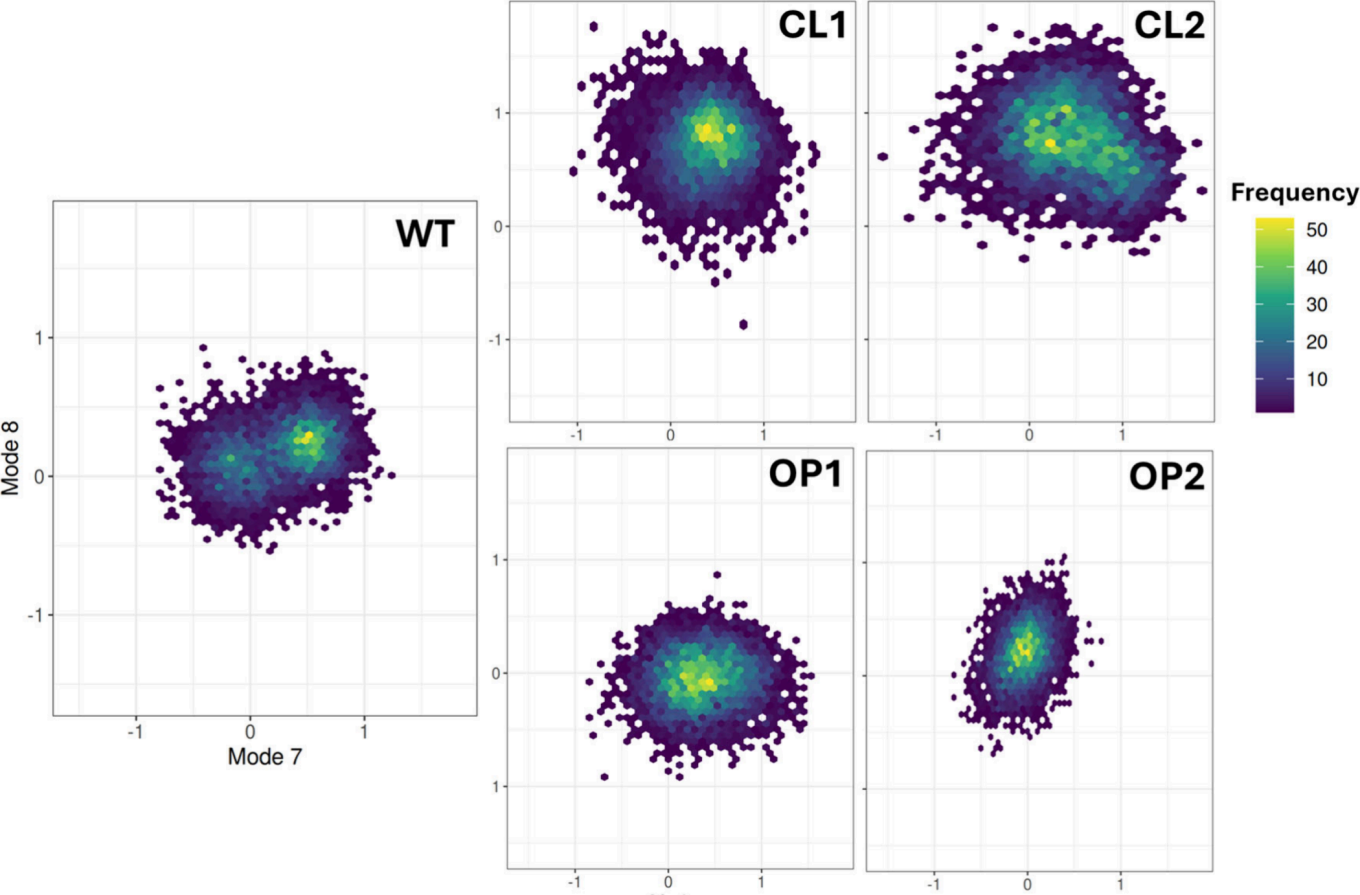
Distribution of conformational states sampled during MD projections onto normal modes 7 and 8 calculated for the WT system. The figure shows the trajectory projections of MD-derived frames onto normal modes 7 and 8 for each design and the WT enzyme.

To further understand the conformational variability, each concatenated trajectory was projected onto the lowest frequency normal modes (Figure 7). In contrast to the wild-type (WT) protein, which exhibits two distinct populations of frames, all engineered designs exhibit a singular densely populated region. Notably, with the exception of OP2, the designs also explore wider regions within collective coordinate space along mode 7. Once again, both closed and open designs show consistent behavior despite sequence differences, confirming that the displaced structure considered for the design procedure is the determinant factor that gives rise to similar dynamic behaviors.

### Understanding the Effects of Mutations through the Network of Frustrated Contacts

**Figure 8:**
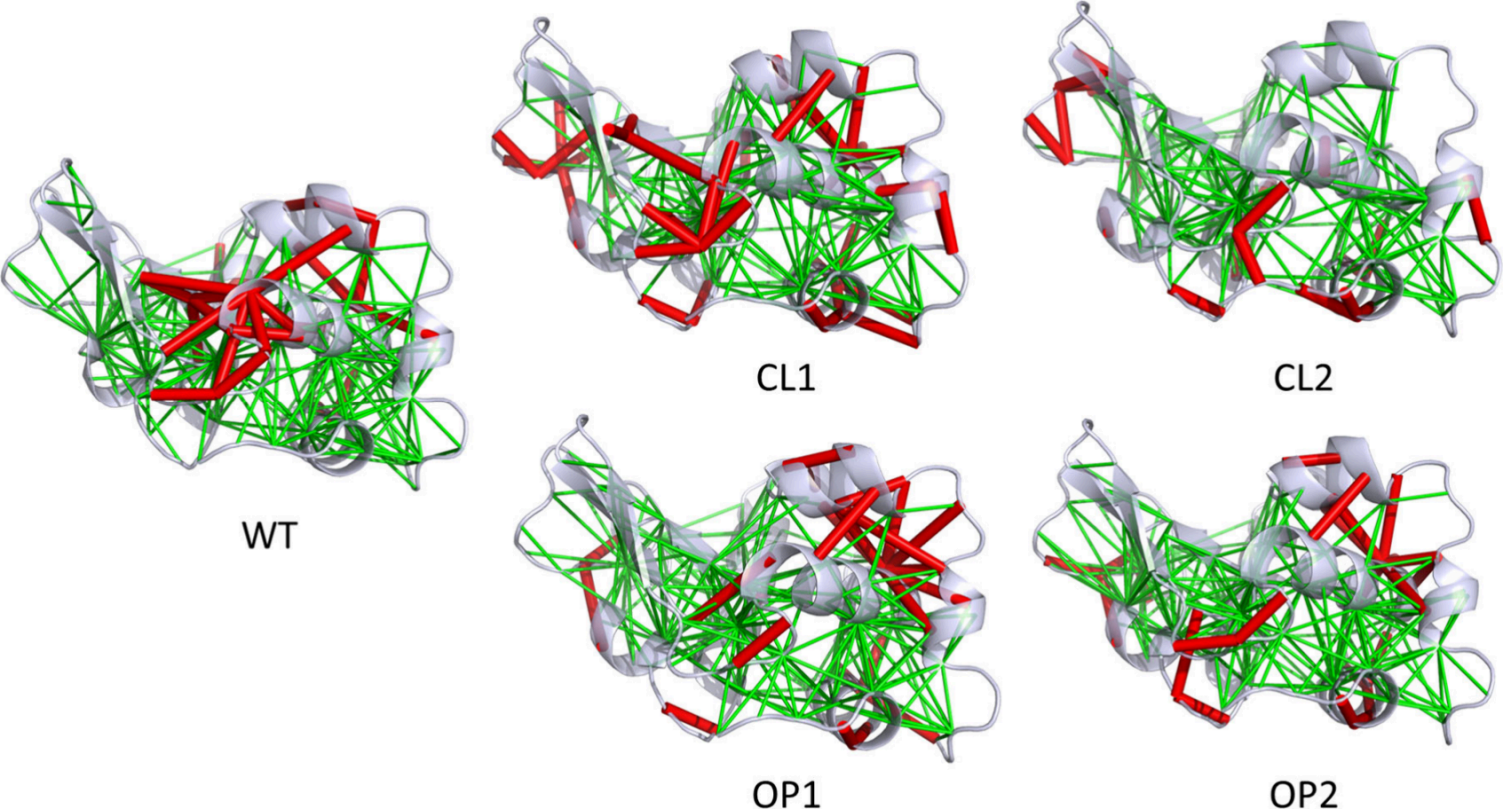
Local frustration analysis. Visualization of the frustration networks mapped onto each construct. Minimally and highly frustrated contacts are represented in green and red tubes, respectively.

We conducted a local frustration analysis to determine how favorable contacts and energetic conflicts are spread in the global structure of the WT and mutated forms (Figure 8). To this end, we conducted a mutational frustration analysis where the effect of sequence perturbations can be evaluated.^14^ To enable fair comparisons, calculations were performed on all constructs modeled taking the WT form as reference, without including NMA related displacements.

The WT lysozyme central cores of each domain (most noticeably on the alpha domain) are minimally frustrated, as commonly observed for other globular proteins.^15^ This feature was preserved in all designs. We also observed a dense region of highly frustrated interactions in the connection of both domains, but no energetic conflicts were observed in the beta domain. Regarding this particular domain, we noticed highly frustrated contacts on the CL constructs, indicating that the reference conformation (partially open), while stable in the WT form, becomes less favorable upon the mutations introduced. On the other hand, in the OP constructs, no energetic conflicts were noticed in the beta domain. In addition, the interface between both domains became less frustrated in both OP constructs, which might be correlated with their increased ability to explore more open states. The analysis of the residue-wise energy contributions of the constructs also reveals residues that are suboptimal compared to the WT, corroborating the analysis of frustrated contacts (Figure S2).

## Conclusion

In conclusion, the method presented in this study offers a promising avenue for engineering protein dynamics through a combination of NMA and structure-based protein engineering. By displacing protein structures along selected normal modes and subsequently optimizing them through mutation, MELT enables the selection of conformational states that differ from those observed for the WT system. Through our investigation using hen-egg lysozyme as a case study, we have demonstrated the efficacy of MELT in sculpting protein energy landscapes and controlling relevant collective coordinates, such as the opening and closing motions of the protein structure in MD simulations. Despite being demonstrated using NMA, the method could theoretically be used with the variations observed in data sets of experimental structures, representative structures derived from MD simulations.

Our results show that designs derived from both open and closed structures exhibit distinct but predictable behaviors in terms of conformational dynamics. Closed designs tend to explore states with lower distances between key residues involved in substrate binding, while open designs skew the distribution toward more open conformations. Furthermore, MELT-generated designs consistently display similar fluctuation patterns despite sequence variations, indicating the robustness of the method in creating desired dynamic behaviors.

Additionally, our analysis reveals the impact of mutations on the global structure and local interactions within the protein. Through frustration analysis and cavity analysis, we observe how mutations introduced by MELT can alter the dynamics of key functional regions, such as the active-site cavity. Future studies will be conducted focusing on optimization of the beta domain, as the pattern of frustrated interactions in this region appears to dictate the overall conformational state (Figure 8).

Here, the designs are created using Rosetta’s ref2015_cart energy function, using structures displaced along normal modes calculated using elastic networks and are evaluated in MD simulations using an unrelated force field. Despite the fact that the MD simulations using the Amber99Sb-Ildn force field corroborates the motion patterns expected from the designs, experimental methods such as FRET (Fluorescence Resonance Energy Transfer) can also be explored in the future. Another investigation, yet to be carried out, is the demonstration that proteins with different folds can be engineered in a similar way. Moreover, our results show the impact of the conformational state submitted to the pyRosetta design procedure on the produced sequences and their dynamical properties, a feature often overlooked in other protein engineering studies using this routine.^16−18^

There is plenty of room for future developments. In order to, at least partially, avoid the structural distortions due to the linear displacements along normal modes, one can use other approaches based on recent approaches allowing nonlinear displacements.^19,20^ In addition, the choice of specific modes for describing a conformational transition of interest is crucial. It may include one or more motions known to explain a given phenomenon (i.e., active-site opening, channel gating). Once experimental data is available, one can select modes that better describe the conformational changes related to function. In our proof-of-concept study, we had prior knowledge that lysozyme mode 7 describes a breathing motion related to active site opening.^21^ We intend to include in MELT an algorithm to select linear combinations of normal modes that better describe a given phenomenon.

Overall, the MELT approach represents a significant advancement in the field of protein engineering, offering a powerful tool for modulating protein dynamics with potential applications in various fields, including enzyme design for industrial purposes, drug development, and biomaterials engineering. Another potential application is in the design of vaccines and other immunogens, where constructs engineered with MELT could favor states where the relevant epitopes are more exposed, facilitating efforts such as the stabilization of SARS-CoV-2 SPIKE protein’s conformational dynamics.^22−24^ The same could be done for therapeutic proteins such as antibodies, and relevant conformations of complementarity determining regions could be stabilized.

Moving forward, further optimization and refinement of the method could unlock even greater potential for tailored manipulation of protein energy landscapes and dynamics, paving the way for the design of proteins with novel functionalities and enhanced properties.

## Methods

### MELT Procedure

The crystal structure of hen-egg lysozyme (PDB code: 2lzt)^25^ was selected as a case study. Normal mode calculations were carried out using R^26^ with the Bio3d library.^27^ The AAENM atomistic elastic network model was employed to describe the interactions.^28^

**Figure 9:**
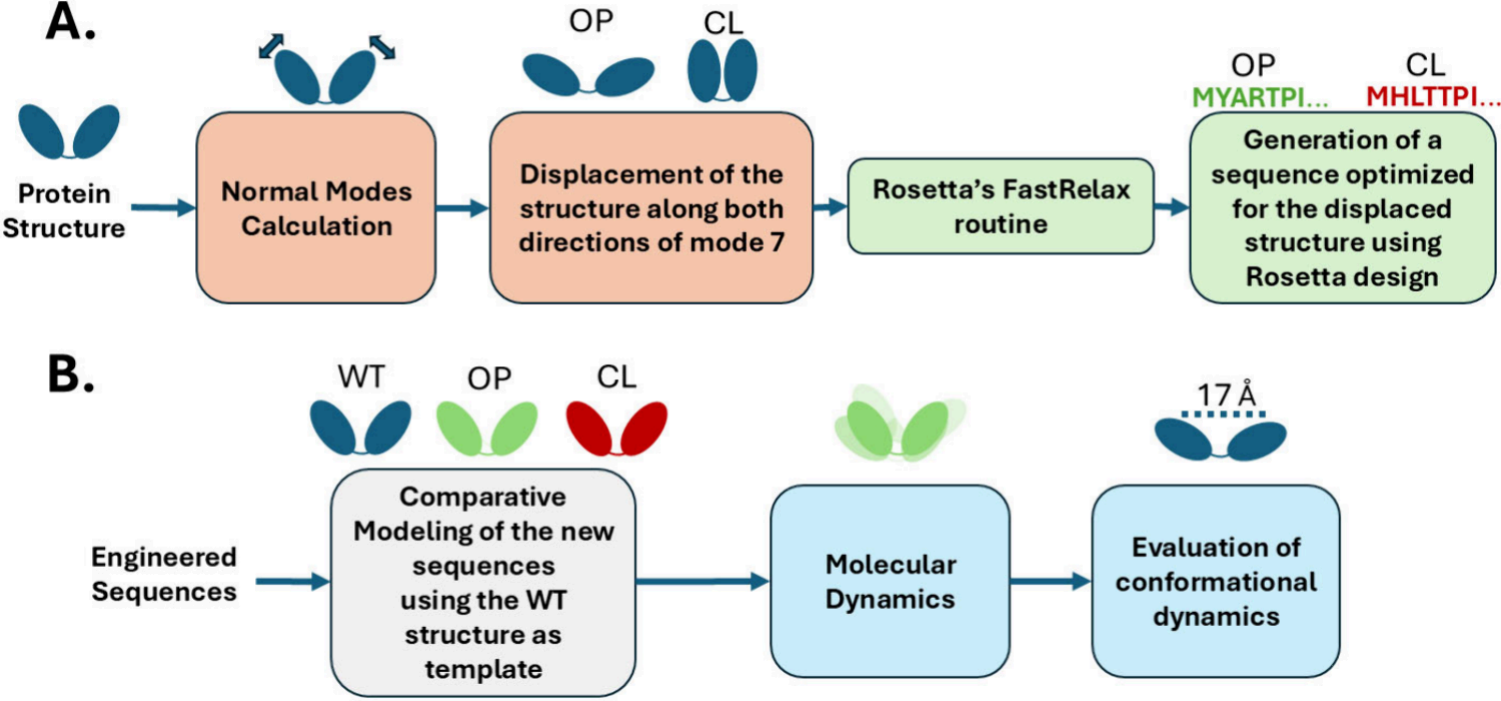
Schematic representation of the MELT procedure and evaluation. (A) MELT procedure: The crystal structure of hen egg-white lysozyme (PDB code: 2lzt) was used as a starting point. NMA was performed to identify the lowest frequency mode (mode 7), which corresponds to a hinge-bending motion. The structure was displaced along both directions of mode 7 (open and closed conformers). Each displaced structure was relaxed and mutated using Rosetta design to generate new sequences (OP1, OP2, CL1, CL2). (B) Evaluation: Engineered sequences were modeled using the WT lysozyme structure as template. MD simulations were performed to evaluate the conformational dynamics of the engineered proteins and compare them to the WT.

Following NM calculations, the reference structure was displaced along both directions of the lowest frequency mode (mode 7) with a magnification factor of 2 Å, which describes a hinge-bending motion related to substrate entrance.^29^ This procedure yielded both an “open” and a “closed” conformer. These structures were relaxed using pyRosetta’s^30^ FastRelax protocol and then mutated using pyRosetta’s design routine, which uses Monte Carlo calculations to select the most energetically favorable residue at each position.^31^ The side chains of residues neighboring each mutated amino acid (10 Å radius) are optimized to better accommodate the mutation. These procedures were performed with a fixed backbone using Rosetta’s ref2015_cart energy function.^32^ Two designs were produced taking as reference the displaced open structure (namely OP1 and OP2) and other two based on the closed structure (CL1 and CL2). The overall procedure for design and evaluation can be seen in Figure 9.

### Structure Prediction and Assessment

The effects of the design procedure on the conformational dynamics of the newly created proteins were evaluated through MD simulations. In order to guarantee a similar initial set of coordinates for the MD simulations and decrease biases related to different initial conformations, all structures were modeled using the WT lysozyme (PDB code 2lzt) as a template using Modeller 10.4.^33^ All generated models were refined through an optimization protocol comprising 300 iterations of energy minimization using Modeller’s variable target function method with conjugate gradient, followed by Modeller’s simulated annealing routine. The optimization process was iterated at least twice and continued until the molpdf (Modeller probability density function) returned values greater than 1 × 10^–6^. The pairwise interaction energies between residues in each design were calculated using INTAA.^34^

### Molecular Dynamics

The systems were immersed in dodecahedral water boxes, filled with the TIP3P water model. The box sizes were chosen to ensure the protein remained 12 Å from the boundaries. The systems were neutralized by adding enough ions Na^+^ or Cl^–^. Energy minimization was then performed on all systems using the steepest descent method, with a limit of 100,000 steps and a convergence threshold of 0.01 kJ mol^–1^ nm^–1^. After minimization, each system was split into two replicas. For the heating phase, distinct random seeds were used to assign velocities to each replica, which were then heated to 310 K over the first 0.5 ns of a 1 ns molecular dynamics (MD) run under the NVT ensemble. Temperature control was achieved using the v-rescale thermostat,^35^ with position restraints applied during heating. This was followed by two consecutive 1.5 ns NPT runs for each replica—one with position restraints and one without—both using the Berendsen barostat^36^ for pressure regulation. Finally, 500 ns production simulations were conducted for each of the three replicas, using the v-rescale thermostat and the Parrinello–Rahman barostat.^37^ All steps were performed using Gromacs 2018^38^ and the Amber-ff99sb-ildn force field.^39^ Hydrogen-heavy atom bonds were constrained. A 2 fs time step was used, and snapshots recorded every 20 ps. Particle Mesh Ewald (PME) handled electrostatics, with Fourier spacing set to 0.12 nm and PME order of 4. The Ewald-shifted direct potential cutoff was set at 1 × 10^–5^.^40^ van der Waals interactions were limited by a 1 nm cutoff.

The root-mean-square fluctuations (RMSF), energetic contributions and cavities of the centroid structures were calculated from the MD frames. The cavities were calculated using the DogSiteScorer server,^41^ and the distance between the Cα atoms of residue 47 and residue 109 was used to describe the opening and closing of the enzyme along mode 7, since they are on opposite sides of the substrate-binding cleft and move in relation to one another when displaced along the mode.

### Local Frustration Analysis

Local Frustration Analysis was conducted with the FrustratometerR package.^42^ Energetic frustration is obtained by comparing the native state interactions with the generated decoy states by mutating each residue. Interactions are defined as highly or minimally - frustrated by comparing frustration energy values with decoy states, as described previously.^14^

## Data Availability

The MELT procedure, code, and documentation is available at https://github.com/izzetbiophysicist/MELT. Molecular dynamics inputs and trajectories are available at https://zenodo.org/records/13951727.

## Supporting Information Available

Figure S1. Cavity analysis of each design. Figure S2. Interaction energy contributions of each design’s residues. (PDF)

## Abbreviations

MELT: Mutational Energy Landscape Trap
NMA: Normal Mode Analysis
MD: Molecular Dynamics
WT: Wild-Type
RMSF: Root Mean Square Fluctuation
RMSD: Root Mean Square Deviation
CL: Closed (as used in designs CL1, CL2, i.e., Closed 1 and Closed 2)
OP: Open (as used in designs OP1, OP2, i.e., Open 1 and Open 2)
Δ*G*_fold_: Gibbs Free Energy of Folding
PDB: Protein Data Bank
