# Supplementary material for: Engineering Protein Dynamics through Mutational Energy Landscape Traps
Source: J Chem Inf Model. 2025 Jan 8;65(2):517–27. doi: 10.1021/acs.jcim.4c01928 (PMC11776041; doi:10.1021/acs.jcim.4c01928)
Supplement: Supplementary file 1 — ci4c01928_si_001.pdf [file ci4c01928_si_001.pdf]

Supplementary material

Engineering protein dynamics through Mutational Energy Landscape Traps

Lucas de Almeida Machado[1,2], João Sartori[2,4], Paula Fernandes da Costa Franklin[2,4], Mauricio G.S. Costa[3,4,*], Ana Carolina Ramos Guimarães[2,4,*]

1. Instituto Nacional de Saúde da Mulher, da Criança e do Adolescente – Fiocruz, Rio de Janeiro, Brazil. 22250-020
2. Laboratório de Genômica Aplicada e Bioinovações - Instituto Oswaldo Cruz/Fiocruz, Rio de Janeiro, Brazil 21040−900
3. Programa de Computação Científica Scientific Computing Program – Fiocruz, Rio de Janeiro, Brazil. 21040-900
4. Programa de Pós-Graduação em Biologia Computacional e Sistemas - Instituto Oswaldo Cruz/Fiocruz, Rio de Janeiro, Brazil 21040−900

* The article was jointly directed by Costa, M. G. S and Guimarães, A. C. R.

Corresponding Author: Costa, M. G. S - mauricio.costa@fiocruz.br

Keywords: Protein engineering, Protein dynamics, Normal Mode Analysis, Molecular dynamics, Energy landscape

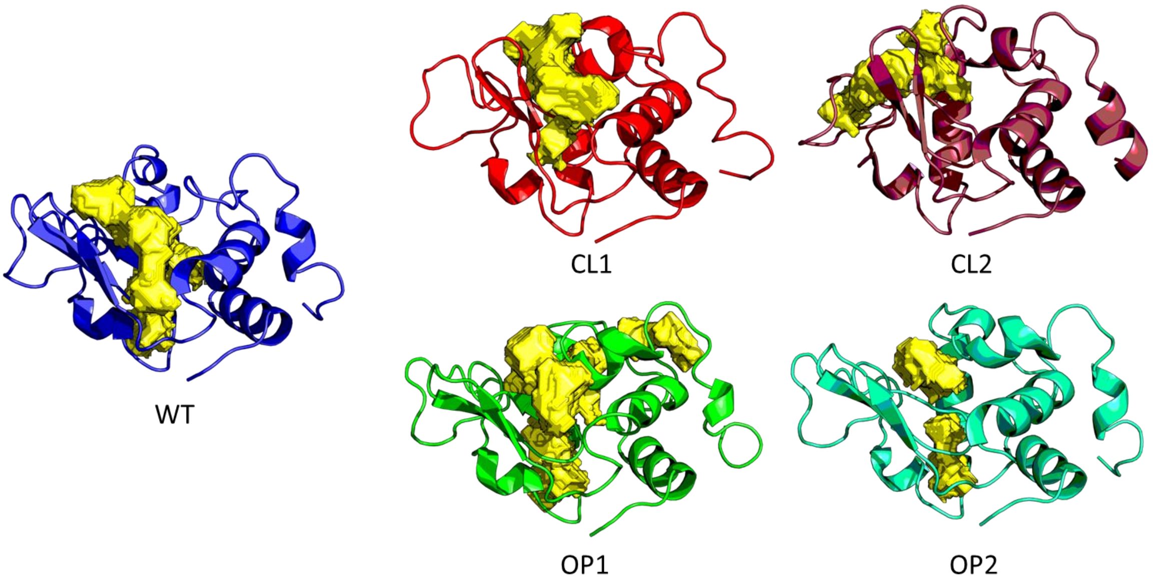

Supplementary Figure 1. Cavity analysis of each design. The shape of the cavities detected overlapping with the active site (yellow) are shown for the MD-derived centroid structure of each design. In some structures, the cavity is further divided into smaller cavities, it is also possible to see overall differences in shape and depth. Analysis of the cavities of the centroid structures derived from the MD of each design showed very distinct patterns in each of the cases. OP1 and OP2 resemble some aspects of the WT cavity, their cavities are as deep as the one detected in WT, however in the centroid structure from OP2 it is fragmented into two smaller cavities. CL1 and CL2 on the other hand lose the depth of the main cavity, and the volume is concentrated in the outermost part. Inspection of the centroid structures also highlights their difference when it comes to compactness, explaining the different shapes of the cavity, the centroids of the closed designs (specially CL2) is way more closed than the open designs (as seen in the MD analysis), constraining the cavity. Since these cavities are approximately overlapped with the substrate binding cleft, these results could also suggest that MELT can play a role in manipulating the opening/closing and morphology of cavities of interest. It is important to highlight that the cavities shown here may suffer from differences in their residues, which can affect their volumes, for instance, OP2 has a much more constrained cavity despite being a more open structure than the WT.

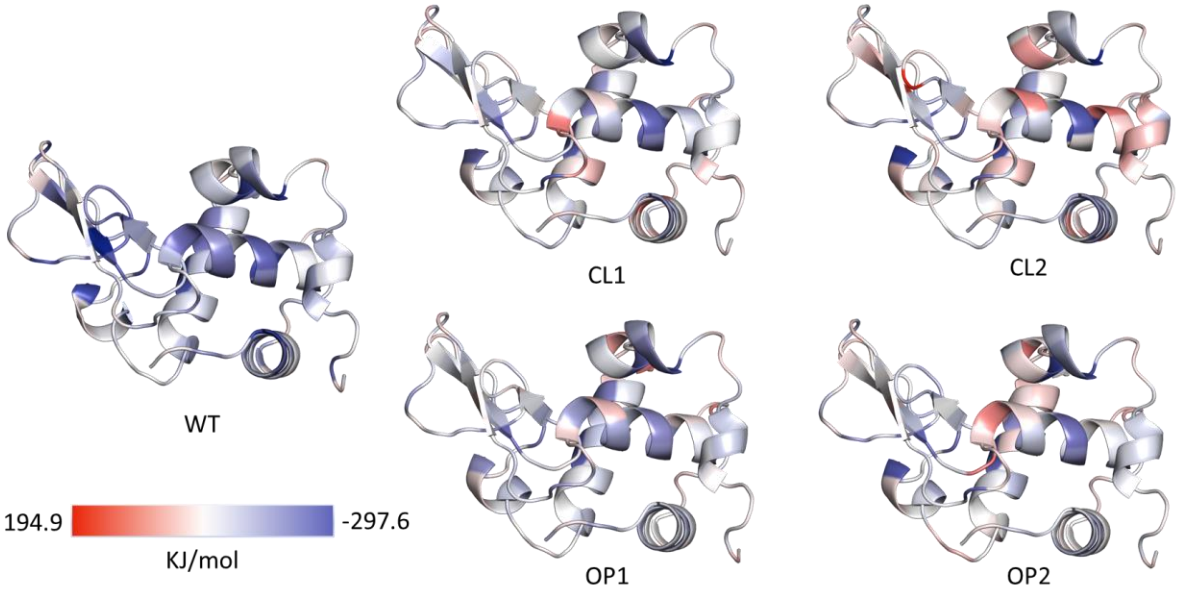

Supplementary Figure 2. Interaction energy contributions of each design's residues**.** Analysis of the energy contributions using INTAA server, the forcefield amber99 and water-like environment (OBC-II). Interestingly, the WT enzyme has less unfavorable contacts than any of the designs, which contrasts with the frustration analysis and CL2 is the design with the most residues with positive energy contribution values.
